# Efficient Escorting Strategy for Aggregation-Prone Notch EGF Repeats with Sparcl1

**DOI:** 10.3390/molecules29051031

**Published:** 2024-02-27

**Authors:** Yuji Kondo, Yuxin Li, Tetsuya Okajima

**Affiliations:** 1 Department of Molecular Biochemistry, Nagoya University Graduate School of Medicine, 65 Tsurumai-cho, Showa-ku, Nagoya 466-8550, Japan; kondoy@med.nagoya-u.ac.jp (Y.K.); li.yuxin.p2@s.mail.nagoya-u.ac.jp (Y.L.); 2 Institute for Glyco-core Research (iGCORE), Nagoya University, Furo-cho, Chikusa-ku, Nagoya 464-8601, Japan

**Keywords:** Notch1, Sparcl1, secretion

## Abstract

Epidermal growth factor (EGF) repeats are present in various proteins and form well-defined structures with three disulfide bonds. One representative protein is the Notch receptor. Each EGF repeat contains unique atypical *O*-linked glycans, such as *O*-linked N-acetylglucosamine (*O*-GlcNAc). To generate a monoclonal antibody against the *O*-GlcNAc moiety in mouse Notch1, we expressed the recombinant C-terminal His_6_-tagged Notch1 EGF14-15 protein in HEK293T cells to prepare the immunogen. Most of the proteins were not secreted and showed higher molecular weight ladders in the cell lysate, suggesting protein aggregation. To overcome this issue, we fused Sparcl1 as an extracellular escorting tag to the N-terminus of Notch1 EGF14-15. The fusion protein was efficiently secreted extracellularly without protein aggregates in the lysates. Following PreScission protease treatment, Notch1 EGF14-15 was efficiently released from the escorting tag. Notch1 EGF14-15 prepared using this method was indeed *O*-GlcNAcylated. The optimal length of the escorting tag was determined by generating deletion mutants to improve the extracellular secretion of EGF14-15. Hence, a large amount of EGF14-15 was successfully prepared from the culture supernatant of HEK293T cells, which were otherwise prone to aggregation.

## 1. Introduction

The epidermal growth factor (EGF) domain is a basic protein module unique to metazoans consisting of 30–40 amino acids and is defined by six conserved cysteine residues that form three pairs of disulfide bonds [1]. In addition, EGF domains undergo three atypical *O*-glycosylations, namely *O*-fucosylation, *O*-glucosylation, and *O*-GlcNAcylation, according to their consensus sequences in each EGF domain, which are mediated by specific glycosyltransferases [2]. EGF domain-specific *O*-fucosylation, *O*-glucosylation, and *O*-GlcNAcylation are catalyzed by POFUT1, POGLUT1, and EOGT, respectively [1,2]. These glycosylations play important roles in proper protein folding and in traversing the secretory pathways designated for the plasma membrane or extracellular spaces [2,3]. The Notch1 receptor has 36 repeated EGF domains that comprise most of the extracellular domain and undergoe the aforementioned atypical *O*-glycosylations of the EGF domains [1]. Among the 36 EGF domains, 22 have a consensus sequence for *O*-GlcNAc modification [4,5]. Using glycoproteomic analysis, 10 of the 22 EGF domains were found to be *O*-GlcNAcylated in recombinant mouse Notch1 prepared from the culture medium of transiently expressed HEK293T cells [4]. This suggests that not only the consensus sequence of *O*-GlcNAcylation but also additional factors contribute to *O*-GlcNAcylation. While targeted inactivation of *Pofut1* or *Poglut1* in mice demonstrates lethal effects on the embryo at midgestation [6,7], the inactivation of *Eogt* does not affect embryonic development and results in normal growth and fertility [8]. Given that Notch *O*-glycosylation plays a critical role in embryonic development by facilitating ligand binding [9], the unimpaired development of *Eogt* null mice may be explained by cell type-restricted expression of *Eogt*, which has not been reported elsewhere. Therefore, it is of fundamental interest to monitor the levels of *Eogt* expression and *O*-GlcNAcylation in mouse Notch1 in appropriate tissues or cell types. However, probes that specifically detect *O*-GlcNAcylated Notch1 are not available, and their development is currently under investigation.

To generate antibodies against *O*-GlcNAcylated EGF domains in Notch1, a significant amount of the recombinant protein must be prepared as an immunogen. The quality control (QC) system in the endoplasmic reticulum (ER) plays a critical role in promoting efficient extracellular secretion of proteins by inducing ER chaperones and COP-II vesicle components, global suppression of protein translation, and ER-associated protein degradation (ERAD). Chemical chaperones also facilitate ER quality control systems [10]. Protein design engineering can also help improve the extracellular secretion of recombinant proteins. This includes codon optimization, an appropriate signal sequence, and the fusion of a known actively secreted protein tag [11].

Here, we report a unique strategy for efficiently secreting aggregation-prone EGF domains extracellularly via mSparcl1 fusion at the N-terminus of the EGF domains.

## 2. Results

### 2.1. Mouse Notch1 Fragments Containing EGF14-16 Tended to Aggregate

**Figure 1:**
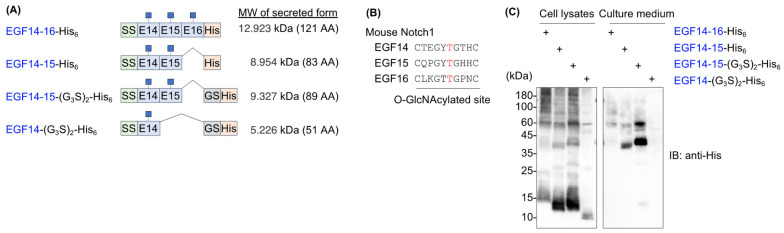
Mouse Notch1 EGF14-16 protein is poorly secreted from HEK293T cells. (**A**) Schematic protein domain structures of C-terminal His-tagged EGF repeats used for forced expression in HEK293T cells. (G_3_S)_2_, glycine–serine linker; AA, amino acid. (**B**) Consensus sequence for *O*-GlcNAcylation by EOGT in the indicated EGF repeats of mouse Notch1. Residues marked in red are the site of *O*-GlcNAc modification. (**C**) Immunoblotting of cell lysates and culture medium from transiently transfected HEK293T cells. Anti-His antibody was used for the analysis.

To generate recombinant proteins bearing *O*-GlcNAc modifications, consecutive EGF14–16 domains derived from mouse Notch1 were selected because these domains possess a consensus sequence for *O*-GlcNAcylation. Furthermore, glycoproteomics data confirmed the *O*-GlcNAc modification [4]. To secrete the EGF domains, they were fused with an IL-2 signal sequence at the N-terminus and a hexa-histidine tag at the C-terminus (EGF14-16-His_6_). We also generated additional constructs truncated at different EGF domains or with a glycine–serine linker inserted between the EGF domain and the hexa-histidine tag (hereafter, EGF14-15-His_6_, EGF14-15-(G_3_S)_2_-His_6_, and EGF14-(G_3_S)_2_-His_6_) (Figure 1A,B).

When these constructs were transiently expressed in HEK293T cells, EGF14-16-His_6_ was barely detectable in the culture medium. Most proteins remained in the cell lysates and showed high molecular weight smears, suggesting protein aggregation (Figure 1C). EGF14-15-His_6_, a truncated version of EGF14-16-His_6_, slightly improved secretion; however, most proteins remained in the lysates. Although the glycine–serine linker inserted into EGF14-15-His_6_ (EGF14-15-(G_3_S)_2_-His_6_) greatly improved its secretion, high molecular weight smears were still evident in the lysate. We could not further narrow down the EGF domain responsible for aggregation because EGF14-(G_3_S)_2_-His_6_ was barely detectable in either the culture medium or cell lysates (Figure 1C). The low level of protein detection of EGF14-(G_3_S)_2_-His_6_ is probably due to inefficient retention on the PVDF membrane during the transfer of the protein.

### 2.2. Chemical Chaperone 4-PBA Does Not Enhance the Secretion of EGF14-15-(G_3_S)_2_-His_6_

**Figure 2:**
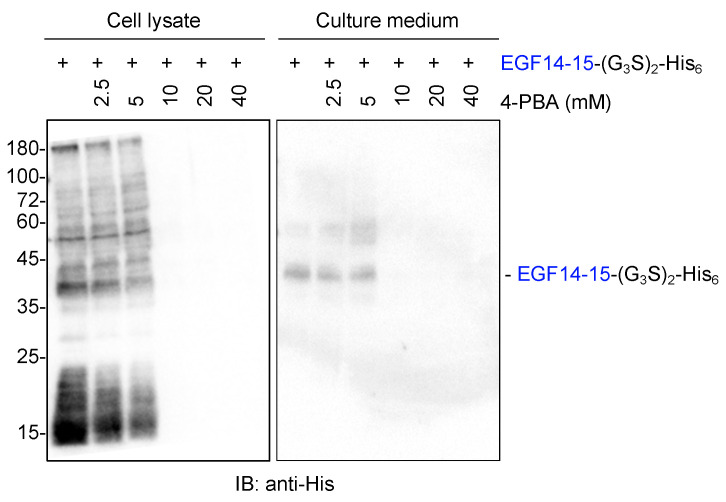
The chemical chaperone 4-PBA does not promote the secretion of EGF14-15-(G_3_S)_2_-His_6_. Immunoblotting of cell lysates and culture medium from transiently transfected HEK293T cells with anti-His antibody. Cells were transiently transfected with a plasmid-encoding Notch1 EGF14-15-(G_3_S)_2_-His_6_ for 6 h, and the culture medium was replaced with serum-free Opti-MEM1 supplemented with the indicated concentration of 4-PBA. Cell lysates and culture media were analyzed three days after transfection.

To improve the extracellular secretion of proteins, transiently transfected cells were simultaneously treated with 4-phenylbutyric acid (4-PBA), a chemical chaperone approved by the FDA [10,12,13]. However, 4-PBA treatment did not enhance EGF14-15-(G_3_S)_2_-His_6_ secretion but rather gradually reduced the global protein expression of EGF14-15-(G_3_S)_2_-His_6_, probably due to the suppression of protein translation (Figure 2).

### 2.3. Sparcl1 Fusion Facilitates Extracellular Secretion of EGF14-15-(G_3_S)_2_-His_6_

**Figure 3:**
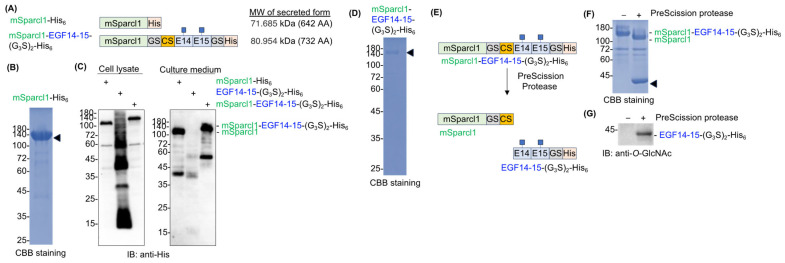
Sparcl1 fusion facilitates extracellular secretion of Notch1 EGF14-15. (**A**) Schematic protein domain structures of mSparcl1-His_6_ and mSparcl1-EGF14-15-(G_3_S)_2_-His_6_ used for forced expression in HEK293T cells. (G_3_S)_2_, glycine–serine linker; CS, cleavage site of the PreScission protease. (**B**) CBB staining of mSparcl1-His6 secreted from HEK293T cells. mSparcl1-His_6_ (arrowhead) was purified from the culture medium using Ni-NTA agarose. (**C**) Immunoblotting with anti-His antibody for analysis of cell lysates and culture medium from transiently transfected HEK293T cells. Plasmids encoding mSparcl1-His_6_, EGF14-15-(G_3_S)_2_-His_6_, or mSparcl1-EGF14-15-(G_3_S)_2_-His_6_ were transiently transfected into HEK293T cells, and the culture medium was replaced with serum-free Opti-MEM1. Cell lysates and culture medium were analyzed three days after transfection. While EGF14-15-(G_3_S)_2_-His_6_ was barely secreted, secretion of the mSparcl1 fusion was significantly enhanced. (**D**) CBB staining of mSparcl1-EGF14-15-(G_3_S)_2_-His_6_ secreted from HEK293T cells. mSparcl1-EGF14-15-(G_3_S)_2_-His_6_ (arrowhead) was purified from the culture medium using Ni-NTA agarose. (**E**) Schematic representation of mSparcl1-EGF14-15-(G_3_S)_2_-His_6_ cleavage by PreScission protease. PreScission protease released EGF14-15-(G_3_S)_2_-His_6_ from the mSparcl1 tag. (**F**) CBB staining of mSparcl1-EGF14-15-(G_3_S)_2_-His_6_ cleaved by PreScission protease. The position of EGF14-15-(G_3_S)_2_-His_6_ is indicated by an arrowhead. (**G**) Immunoblotting with an anti-*O*-GlcNAc antibody to analyze the presence of *O*-GlcNAc on prepared EGF14-15-(G_3_S)_2_-His_6_.

While producing various recombinant secretory proteins in HEK293T cells in the laboratory, we obtained an unexpectedly high yield of mouse Sparcl1 with a C-terminal His tag (mSparcl1-His_6_) from the culture medium (Figure 3A,B). Approximately 1 mg of mSparcl1-His_6_ was recovered from 60 mL of the culture medium. Sparcl1 (SPARC-like 1) is a type I secretory protein (650 amino acids) with three potential *N*-glycosylation sites and has been implicated as a tumor suppressor protein [14,15,16,17]. The protein was initially reported to be specific for blood endothelial cells, but recent single-cell RNA sequencing (scRNA-seq) data in periodontal tissues revealed that it is a distinct cementoblast/cementocyte-specific marker [14,15].

While the specific function of Sparcl1 is yet to be fully elucidated, we aimed to enhance protein secretion by fusing the mouse Sparcl1 protein (mSparlc1) to aggregation-prone EGF14-15-(G_3_S)_2_-His_6_ (Figure 3A). To remove mSparcl1 from EGF14-15-(G_3_S)_2_-His_6_ after purification, a PreScission protease cleavable sequence (LEVLFQ↓GP) was inserted between mSparcl1 and EGF14-15-(G_3_S)_2_-His_6_. While mSparcl1-His_6_ was actively secreted and EGF14-15-(G_3_S)_2_-His_6_ was barely secreted into the culture medium, the fused protein (hereafter, mSparcl1-EGF14-15-(G_3_S)_2_-His_6_) greatly enhanced its extracellular secretion (Figure 3C). Surprisingly, mSparcl1-EGF14-15-(G_3_S)_2_-His_6_ showed a single band in cell lysates, suggesting that EGF14-15 protein aggregation was resolved by the mSparcl1 fusion. As expected, a high protein yield of mSparcl1-EGF14-15-(G_3_S)_2_-His_6_ was observed, which was similar to that of mSparcl1-His_6_ (Figure 3D). Then, purified mSparcl1-EGF14-15-(G_3_S)_2_-His_6_ was incubated with PreScission protease at 4 °C for 16 h to cleave EGF14-15-(G_3_S)_2_-His_6_ from mSparcl1 fusion, and successful cleavage of mSparcl1-EGF14-15-(G_3_S)_2_-His_6_ was observed (Figure 3E,F). The blot was probed with an anti-*O*-GlcNAc antibody, and the presence of *O*-GlcNAc modification on the prepared protein was confirmed (Figure 3G).

To investigate the expression status of aggregation-prone EGF14-15-(G_3_S)_2_-His_6_ in lysates, cell lysates from HEK293T cells transiently transfected with EGF14-15-(G_3_S)_2_-His_6_ were analyzed by native PAGE as well as non-reducing and reducing SDS-PAGE (Supplementary Materials Figure S1A). In all experiments, aggregation-prone EGF14-15-(G_3_S)_2_-His_6_ showed smeared bands, whereas mSparcl1-EGF14-15-(G_3_S)_2_-His_6_ showed a single band, suggesting that EGF14-15-(G_3_S)_2_-His_6_ forms aggregates within cells even in the native state. This finding was further supported by the observation of increased aggresome formation in HEK293T cells transfected with EGF14-15-(G_3_S)_2_-His_6_ compared to empty vector-transfected cells (Supplementary Materials Figure S1B) [16,17]. To identify where EGF14-15-(G_3_S)_2_-His_6_ accumulates in cells, HEK293T cells simultaneously transfected with EGF14-15-(G_3_S)_2_-His_6_ as well as ER, and Golgi markers were immunolabelled with an anti-His antibody. Although the intensity of the His-tagged protein was unexpectedly low, some of the protein co-localized with the ER marker, suggesting ER accumulation of EGF14-15-(G_3_S)_2_-His_6_ (Supplementary Materials Figure S2). Poor immunolabeling with anti-His against aggregation-prone EGF14-15-(G_3_S)_2_-His_6_ may be due to the inaccessibility of the antibody to the hexa-histidine tag in protein aggregates.

### 2.4. Deletion Mutants of mSparcl1 and Their Effects on the Secretion of EGF14-15-(G_3_S)_2_-His_6_

**Figure 4:**
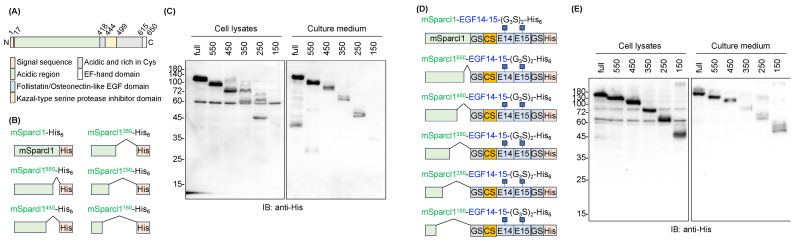
Deletion mutants of mSparcl1 and their effect on assisting Notch1 EGF14-15 secretion. (**A**) Schematic protein domain structures of mouse Sparcl1. (**B**) Schematic protein domain structures of mSparcl1-His_6_ and its C-terminally deleted mutants used for forced expression in HEK293T cells. (**C**) Immunoblotting with an anti-His antibody for analysis of cell lysates and culture medium from transiently transfected HEK293T cells. (**D**) Schematic protein domain structures of mSparcl1-EGF14-15-(G_3_S)_2_-His_6_ and its C-terminally deleted mutants used for forced expression in HEK293T cells. (**E**) Immunoblotting with an anti-His antibody for analysis of cell lysates and culture medium from transiently transfected HEK293T cells.

Since full-length mSparcl1 consists of 650 amino acids with functionally distinct domains [18,19], the effect of truncation in mSparcl1 on the protein secretion of mSparcl1-His_6_ was examined (Figure 4A,B). Full-length and five C-terminal truncated mutants of mSparcl1-His_6_ were independently transfected into HEK293T cells, and protein expression in cell lysates and culture medium was analyzed. Full-length mSparcl1-His_6_, mSparcl1^550^-His_6_, and mSparcl1^450^-His_6_ demonstrated high secretion, whereas shorter mSparcl1-His_6_ constructs exhibited reduced secretion into the culture medium (Figure 4B,C). Similarly, the effect of mSparcl1 truncation on the protein secretion of mSparcl1-EGF14-15-(G_3_S)_2_-His_6_ was examined (Figure 4D). Consistent with the results for mSparcl1-His_6_ (Figure 4C), mSparcl1-EGF14-15-(G_3_S)_2_-His_6_ (full-length), mSparcl1^550^-EGF14-15-(G_3_S)_2_-His_6_, and mSparcl1^450^-EGF14-15-(G_3_S)_2_-His_6_ were secreted efficiently (Figure 4E). Notably, slightly smeared bands were observed in cell lysates from mSparcl1^150^-EGF14-15-(G_3_S)_2_-His_6_, indicating an inadequate enhancement in EGF14-15-(G_3_S)_2_-His_6_ secretion with the shorter mSparcl1 construct.

### 2.5. Purification of Deletion Mutants of mSparcl1-EGF14-15-(G_3_S)_2_-His_6_

**Figure 5:**
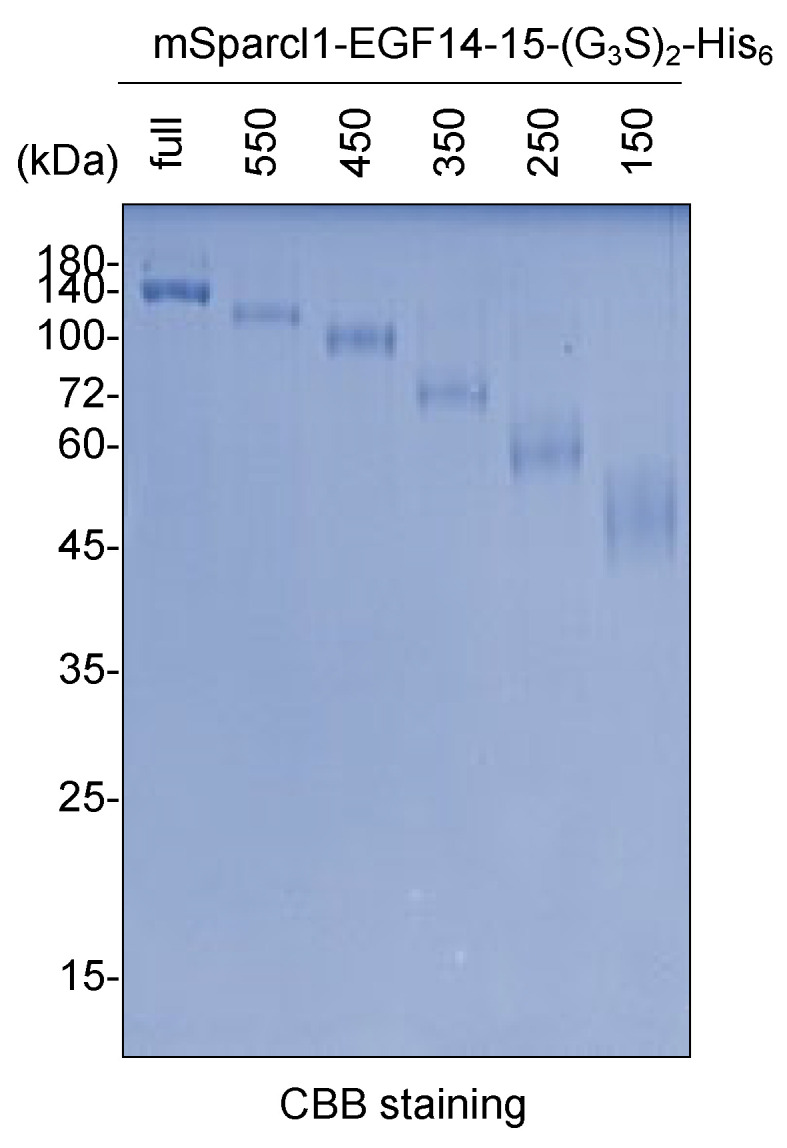
Purification of mSparcl1-EGF14-15-(G_3_S)_2_-His_6_ deletion mutants. CBB staining of mSparcl1-EGF14-15-(G_3_S)_2_-His_6_ and its C-terminal deletion mutants secreted from HEK293T cells. All proteins were purified from the culture medium using Ni-NTA agarose.

The low efficiency of detection of the shorter protein mSparcl1-His_6_ and mSparcl1-EGF14-15-(G_3_S)_2_-His_6_ could be due to the insufficient retention of proteins on the PVDF membrane during immunoblotting (Figure 4C,E). To eliminate this possibility, full-length and five truncated mutants of mSparcl1-EGF14-15-(G_3_S)_2_-His_6_ purified from the culture medium were subjected to CBB staining. Consistent with the immunoblotting data (Figure 4), higher yields of mSparcl1-EGF14-15-(G_3_S)_2_-His_6_ (full-length), mSparcl1^550^-EGF14-15-(G_3_S)_2_-His_6_, and mSparcl1^450^-EGF14-15-(G_3_S)_2_-His_6_ were observed, suggesting that the efficient protein secretion capability of mSparcl1 was attributed to the N-terminal region (1–450) (Figure 5).

### 2.6. Co-Transfection of mSparcl1 Slightly Enhances Secretion of EGF14-15-(G_3_S)_2_-His_6_

**Figure 6:**
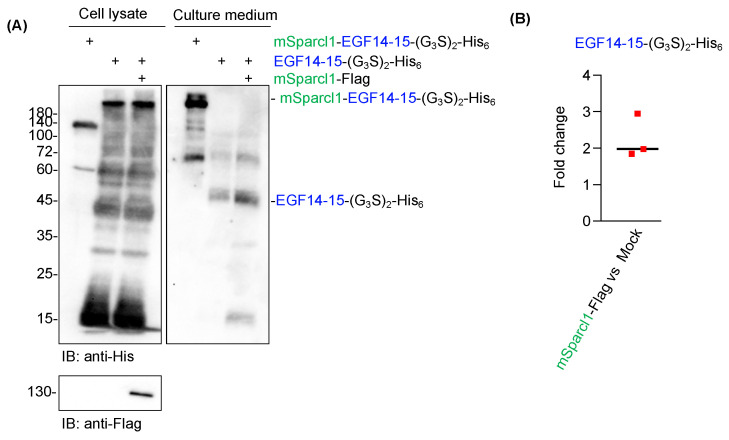
Co-transfection of mSparcl1 slightly increased Notch1 EGF14-15 secretion. (**A**) Immunoblotting with an anti-His antibody to analyze cell lysates and culture medium from transiently transfected HEK293T cells. Cells were transfected with mSparcl1-EGF14-15-(G_3_S)_2_-His_6_, EGF14-15-(G_3_S)_2_-His_6_, or co-transfected with mSparcl1-Flag and EGF14-15-(G_3_S)_2_-His_6_. Blotting with an anti-Flag antibody for the expression of mSparcl1-Flag. (**B**) Fold change of EGF14-15-(G_3_S)_2_-His_6_ secreted with mSparcl1 flag versus mock transfection. Bar; average of fold change from three independent experiments.

Although Sparcl1 is a secretory protein, it has been detected on the cell surface using flow cytometry with a specific antibody [14]. To date, the physiological function of Sparcl1 involves the crosslinking of extracellular matrices [20,21]. However, the reason for the high yields of mSparcl1-His_6_ and mSparcl1-EGF14-15-(G_3_S)_2_-His_6_ remains unknown. To test whether mSparcl1 possesses chaperone-like activity, the co-transfection of plasmids for EGF14-15-(G_3_S)_2_-His_6_ and mSparcl1-Flag was performed into HEK293T cells. Although significant amounts of smeared EGF14-15-(G_3_S)_2_-His_6_ were still detected in cell lysates, secreted levels of EGF14-15-(G_3_S)_2_-His_6_ increased only twofold in mSparcl1-Flag transfected cells (Figure 6A,B). Hence, the effect of Sparcl1 in enhancing protein secretion was primarily attributed to the intrinsic properties of proteins with high secretion rather than chaperone-like activity.

## 3. Discussion

EGF repeats are found in various physiologically important proteins, such as Notch receptors and their cognate ligands [1]. Proper protein folding and post-translational modifications, including glycosylation, in the ER are essential for trafficking EGF domain-containing proteins to their final destination and function properly [2]. Notably, protein glycosylation of EGF domains includes *O*-fucosylation, *O*-glucosylation, and *O*-GlcNAcylation, which are atypical types of *O*-glycosylation, in contrast to the typical mucin-type *O*-glycosylation [2]. Although 22 of the 36 EGF repeats of mouse Notch1 contained a consensus sequence for *O*-GlcNAc modification, only 10 EGF repeats underwent *O*-GlcNAcylation in recombinantly expressed mouse Notch1 in HEK293T cells [4]. In addition, public scRNA-seq data indicate the tissue- or cell-type-specific expression of *O*-glycosyltransferases [22]. This suggests that the level of *O*-glycosylation in EGF repeats may differ among cell types. However, there is a huge knowledge gap in our understanding of the glycoproteome of endogenously expressed proteins in vivo. To address this issue, it is necessary to develop specific probes that can detect specific forms of glycans in EGF repeats. For this purpose, consecutive EGF repeats of EGF14-16 were selected as recombinant proteins that could be used as immunogens. Unexpectedly, C-terminal His_6_ tagged EGF14-16 was barely detected in the culture medium prepared from transiently transfected HEK293T cells, and most of the proteins were not secreted and showed higher molecular weight ladders in the cell lysate, suggesting protein aggregation. Reducing the number of EGF domains to two or one did not improve extracellular secretion (Figure 1C).

We sought methods to facilitate the secretion of aggregation-prone proteins. Initially, 4-PBA was used [10,12,13]. However, we did not observe any improvement in the secretion of EGF14-15-(G_3_S)_2_-His_6_. During the preparation of various recombinant proteins, we encountered an unexpectedly high protein yield of mSparcl1 in the culture medium of transiently transfected cells. Based on this finding, we aimed to evaluate the benefit of fusing the mSparcl1 secretory protein with EGF14-15-(G_3_S)_2_-His_6_. Surprisingly, we observed effective extracellular secretion of the fused protein, with the complete disappearance of aggregates in the cell lysates.

Although the detailed mechanism by which mSparcl1 fusion improves the protein secretion of EGF14-15-(G_3_S)_2_-His_6_ remains elusive, the beneficial effect of mSparcl1 was attributed to the N-terminal domain of mSparcl1 (i.e., 1-450 amino acids). The effect on EGF14-15-(G_3_S)_2_-His_6_ secretion by co-transfection with mSparcl1-Flag, instead of generating mSparcl1 fusion proteins, was limited, making the chaperone-like activity of mSparcl1 unlikely. Thus, similar to fusion partners for the efficient secretion of recombinant proteins in yeast [23,24], the secretability of mSparcl1 may contribute to the increased secretion of aggregation-prone recombinant proteins. To assess the general utility of mSparcl1 fusion for efficient escort function, we tested delta-like homolog 1 as another fusion partner of mSparcl1, as its ectodomain was efficiently secreted from HEK293T cells into the culture medium [25,26]. Whether the mSparcl1 fusion is of general use for aggregation-prone proteins remains to be investigated. 

No prior instances of enhanced secretion through mSparcl1 fusion have been documented, making it crucial to investigate whether the advantageous impact of mSparcl1 on protein secretion extends globally to other secretory proteins. We are also interested in the effect of Sparcl1 fusion on known aggregation-prone proteins in the field of neurodegenerative diseases, such as amyloid β in Alzheimer’s disease, α-synuclein in Parkinson’s disease, huntingtin protein in Huntington’s disease, and SOD1 in amyotrophic lateral sclerosis. This is a future research direction [27,28,29,30]. Sparcl1 is expressed in a variety of cell types, including neurons [31], and genetic mutations in *SPARCL1* are associated with multiple sclerosis and autism spectrum disorders [32,33]. The latter study also showed that the W647R mutation impaired the export of Sparcl1 from the ER [33]. Therefore, the pathophysiological function of Sparcl1 in secretion should be investigated in future studies.

## 4. Materials and Methods

### 4.1. Reagents

Reagents for tissue culture and transfection, DMEM (#SH30002, Cytiva, Marlborough, MA, USA), fetal bovine serum (FBS) (#175012, NICHIREI, Tokyo, Japan), Opti-MEM1 (#22600-134, Gibco, Billings, MT, USA), PEImax (#24885-2, Polysciences Inc., Warrington, PA, USA), and sodium 4-phenylbutyrate (4-PBA) (#O0511, TCI, Tokyo, Japan) were purchased. For SDS-PAGE and immunoblotting, mouse anti-His monoclonal antibody (#652501, Biolegend, San Diego, CA, USA), rat anti-FLAG monoclonal antibody (#637301, Biolegend, USA), HRP-anti-*O*-GlcNAc (#12938, CST, Danvers, MA, USA), CBB-R-250 (#031-17922, FUJIFILM, Tokyo, Japan), cell lysis buffer (10X) (#9803, CST, USA), and cOmplete protease inhibitor cocktail (#11697498001, Roche, Indianapolis, IN, USA) were purchased. For protein purification, Ni-NTA agarose (#143-09763, FUJIFILM, Tokyo, Japan), PreScission protease (#27-0843-01, Cytiva, Marlborough, MA, USA), Amicon filter (#UFC901096, Millipore, Burlington, MA, USA), empty polyprep chromatography columns (#7311550, Bio-Rad, Hercules, CA, USA), HBSS (#084-08965, FUJIFILM, Tokyo, Japan), and imidazole (#095-00015, FUJIFILM, Tokyo, Japan) were purchased.

### 4.2. Plasmids

Notch1, EGF14-16-His_6,_ and EGF14-15-His_6_ expression vectors were constructed using infusion cloning. Briefly, PCR-amplified EGF domains, IL-2 signal sequences, and hexa-histidine tags were simultaneously cloned into the pcDNA3.1(+) plasmid. PCR templates for the EGF domains were generated by Artificial Gene Synthesis (FASMAC, Kanagawa, Japan). EGF14-15-(G_3_S)_2_-His_6_ and EGF14-(G_3_S)_2_-His_6_ expression vectors were constructed by inverse PCR using EGF14-15-His_6_ as the PCR template. The vectors encoding mouse Sparcl1-His_6_ or Sparcl1-Flag were constructed by inserting PCR-amplified full-length mouse Sparcl1 into the pcDNA3.1(+) plasmid. The mSparcl1-EGF14-15-(G_3_S)_2_-His_6_ expression vector was constructed by inverse PCR using mSparcl1-His_6_ as the PCR template. Deletion mutants of mSparcl1 and its EGF14-15-(G_3_S)_2_-His_6_ fused plasmids were constructed by inverse PCR using the parental plasmids as templates. Transfection-grade plasmids were purified from XL10-gold competent cells using the NucleoBond Xtra Midi kit (Takara, Tokyo, Japan).

### 4.3. SDS-PAGE, Immunoblotting, and CBB Staining

HEK293T cells (1 × 10^6^/2 mL) were plated onto six-well plates and transfected with a preformed DNA-PEImax complex consisting of plasmid DNA (3 μg) with PEImax (21 μg) in Opti-MEM1 medium. After 6 h, the culture medium was discarded and replaced with serum-free Opti-MEM1, and the cells were cultured for another 72 h in a 5% CO_2_ incubator at 37 °C. The culture medium was centrifuged at 2500× *g* for 5 min, and the supernatant was mixed with Laemmli SDS sample buffer and boiled at 95 °C for 3 min. To prepare the cell lysates, the cells were detached by mechanical pipetting in chilled PBS and centrifuged for 5 min at 200× *g*. After removal of the supernatant, the cells were lysed with 100 μL of cell lysis buffer plus protease inhibitor cocktail on ice for 10 min. The samples were then centrifuged at 2500× *g* for 5 min at 4 °C, and the supernatant was mixed with Laemmli SDS sample buffer and boiled at 95 °C for 3 min. Protein expression in the cell lysates and culture medium was analyzed using 12% SDS-PAGE. The separated proteins were transferred to the Immobilon-P membrane (Millipore, Burlington, VT, USA). After blocking, the membranes were incubated with antibodies and developed using the ECL system. For CBB staining, the separated gel was stained with CBB-R250 for 30 min and then destained for 30 min. Chemiluminescence on the PVDF membranes and CBB-stained gels was examined using the iBright Imaging System (Thermo, Waltham, MA, USA).

### 4.4. Recombinant Protein Expression and Purification

The recombinant proteins used in this study were prepared in a serum-free culture medium derived from transiently transfected HEK293T cells. Briefly, 1 × 10^7^ cells plated on ten 100 mm dishes were transfected with a preformed DNA-PEImax complex consisting of plasmid DNA (9 μg) with PEImax (63 μg) in Opti-MEM1 media. After 6 h, the culture medium was discarded and replaced with serum-free Opti-MEM1, and the cells were cultured for an additional 72 h in a 5% CO_2_ incubator at 37 °C. The culture medium (approximately 60 mL) was harvested into 50 mL conical tubes and centrifuged at 2500× *g* for 10 min. The supernatant was filtered (0.45 μm) and concentrated for 10 folds using an Amicon 10 kDa cut-off concentrator. The buffer was then replaced with a wash buffer comprising 50 mM Tris-Cl, pH 8.0, 150 mM NaCl, and 1 mM CaCl_2_. Hexa-histidine-tagged proteins were purified by incubating Ni-NTA agarose (500 μL) in the presence of 7.5 mM imidazole overnight at 4 °C with rotation. Ni-NTA agarose packed into an empty column was washed with 10 column volumes (CVs) of wash buffer, and the bound proteins were eluted with five CVs of wash buffer comprising 250 mM imidazole. Absorbance at 280 nm was monitored using an Ultrospec 3000 (Pharmacia Biotech, Piscataway, NJ, USA), and eluted fractions containing proteins were concentrated and further buffer exchanged with HBSS (+) using an Amicon Ultra centrifugal filter (10 kDa cut-off, #UFC501024, Millipore, Burlington, VT, USA). Proteins were quantified using the Pierce BCA Protein Assay Kit (#23225, Thermo, Waltham, MA, USA). Digestion of recombinant proteins with PreScission protease was performed by incubating 1 U of PreScission protease with 100 μg of recombinant protein for 16 h on ice.

### 4.5. Immunofluorescence and Confocal Microscopy

HEK293T cells were transiently transfected simultaneously with three plasmids: ER-mNeonGreen, mCherry-Golgi-7, and EGF14-15-(G_3_S)_2_-His_6_. After 48 h, cells were fixed with 4% paraformaldehyde for 10 min, permeabilized/blocked with 0.05% Triton X-100 in 3% BSA in PBS for 1 h, and immunolabelled with an anti-His antibody (#652501, Biolegend, San Diego, CA, USA) for 1 h. After washing, cells were stained with AlexaFluor680 anti-mouse IgG (#A-21057, ThermoFisher, Waltham, MA, USA). After washing, cells were mounted with DAPI Fluo-romount-G (#0100-20, Southern Biotech, Birmingham, AL, USA). Confocal microscopy images were obtained using a Nikon A1-Rsi confocal microscope equipped with a Plan-Apo 100X/1.40 oil immersion objective.

### 4.6. Aggresome Formation Assay

HEK293T cells were transiently transfected with a plasmid-encoding EGF14-15-(G_3_S)_2_-His_6_. After 48 h, aggresome formation was analyzed using a PROTEOSTAT Aggresome Detection Kit (#ENZ-51035, ENZO, Broomfield, CO, USA) according to the manufacturer’s protocol. Cells transfected with empty plasmid were used as a control.

### 4.7. Native PAGE

HEK293T cells were transiently transfected with either a plasmid-encoding empty vector, EGF14-15-(G_3_S)_2_-His_6_, or mSparcl1-EGF14-15-(G_3_S)_2_-His_6_. After 48 h, cell lysates were processed in either a non-reduced denatured condition, 2-mercaptoethanol reduced denatured condition, or native condition. Except for native samples, samples were boiled at 95 °C for 3 min. Denatured samples were separated by 12% SDS-PAGE and native samples by 7.5% native PAGE (pH 8) [34]. Separated proteins were transferred to a PVDF membrane for normal SDS-PAGE, and protein migration was analyzed with an anti-His antibody.

### 4.8. Data Analysis and Statistics

Statistical tests were performed using Prism 9.1.2 (GraphPad, San Diego, CA, USA). A two-tailed Student’s *t*-test was used to assess the statistical significance of differences between the two groups after confirming that the data met the criteria of normal distribution and equal variance. Differences were considered statistically significant at *p* < 0.05.

## Data Availability

All data will be available upon publication.

## Supplementary Materials

The following supporting information can be downloaded at https://www.mdpi.com/article/10.3390/molecules29051031/s1, Figure S1: EGF14-15-(G_3_S)_2_-His_6_ forms aggregates based on different experimental evidence. A. Proteins in cell lysates from HEK293T cells transiently transfected with EGF14-15-(G_3_S)_2_-His_6_ were analyzed in non-reducing denatured condition, reducing denatured condition, or native condition. The separated proteins were immunoblotted with anti-His antibody. B. HEK293T cells transiently transfected with EGF14-15-(G_3_S)_2_-His_6_ were analyzed for aggresome formation by flow cytometry. Figure S2: EGF14-15-(G_3_S)_2_-His_6_ partially colocalized with ER markers. HEK293T cells transiently transfected with ER-mNeonGreen, mCherry-Golgi-7 and EGF14-15-(G_3_S)_2_-His_6_ were immunolabelled with anti-His antibody. Stained cells were imaged by confocal microscopy using a 100×/1.40 oil immersion objective. Bar, 5 μm.
