# Supplementary material for: Efficient Escorting Strategy for Aggregation-Prone Notch EGF Repeats with Sparcl1
Source: Molecules. 2024 Feb 27;29(5):1031. doi: 10.3390/molecules29051031 (PMC10935072; doi:10.3390/molecules29051031)
Supplement: Supplementary file 1 [file molecules-29-01031-s001.zip › molecules-2874196-supplementary.pdf]

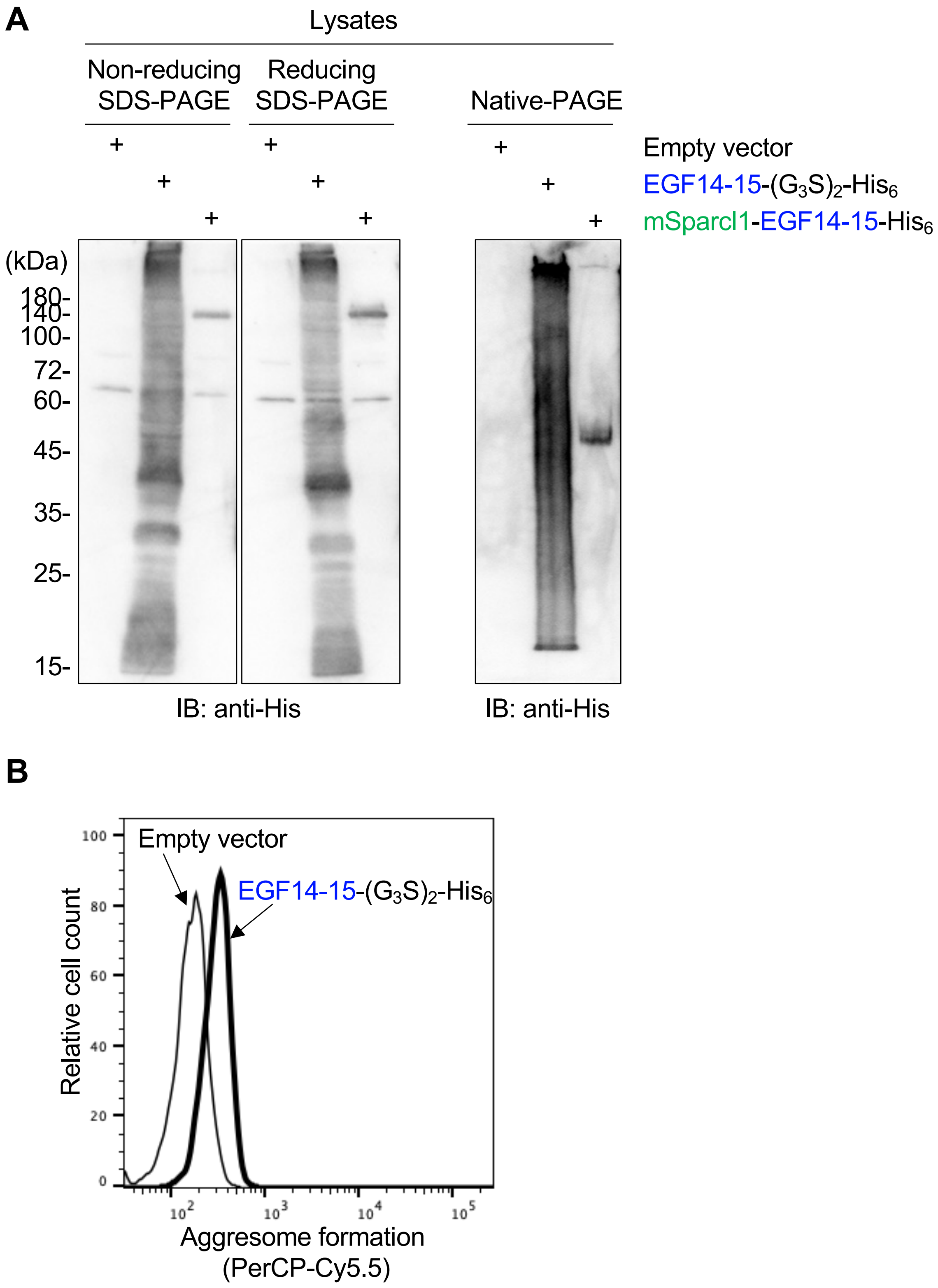


**Supplementary Figure S1. EGF14-15-(G3S)2-His6 forms aggregates based on different experimental evidence.** A. Proteins in cell lysates from HEK293T cells transiently transfected with EGF14-15-$(G_3S)_2$-$His_6$ were analyzed in non-reducing denatured condition, reducing denatured condition, or native condition. The separated proteins were immunoblotted with anti-His antibody. B. HEK293T cells transiently transfected with EGF14-15-$(G_3S)_2$-$His_6$ were analyzed for aggresome formation by flow cytometry.

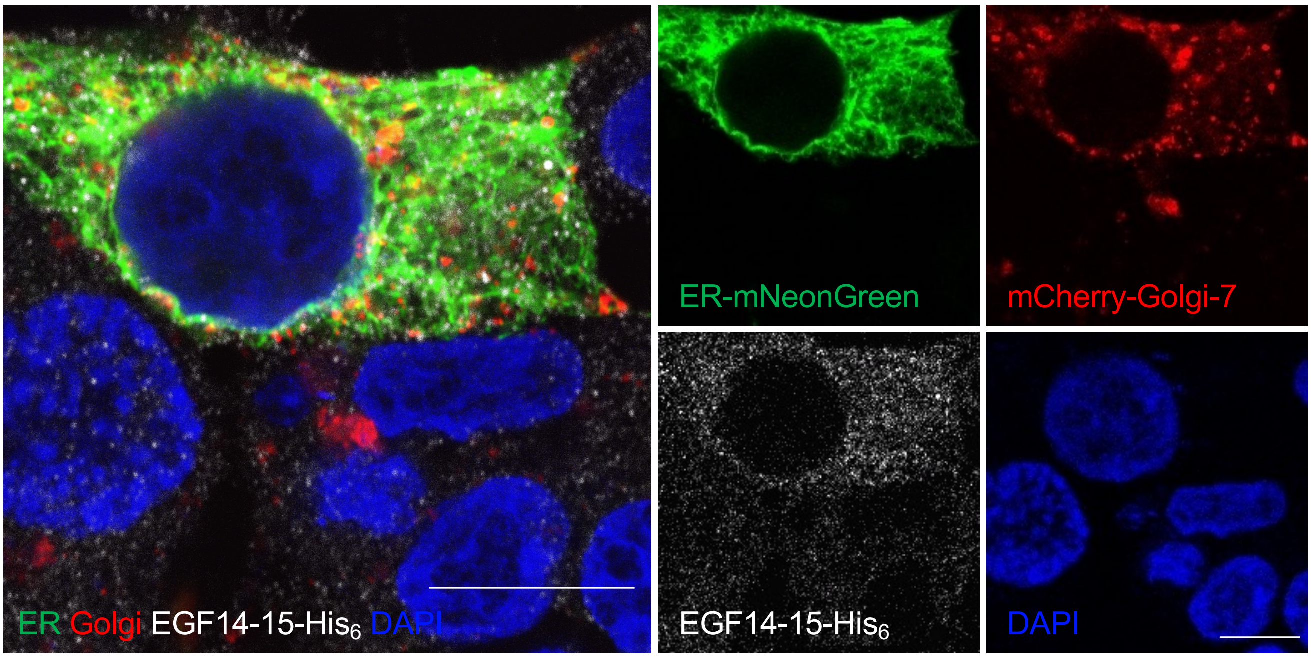


**Supplementary Figure S2. EGF14-15-$(G_3S)_2$-$His_6$ partially colocalized with ER markers.** HEK293T cells transiently transfected with ER-mNeonGreen, mCherry-Golgi-7 and EGF14-15-$(G_3S)_2$-$His_6$ were immunolabelled with anti-His antibody. Stained cells were imaged by confocal microscopy using a 100X/1.40 oil immersion objective. Bar, 5 µm.
